# The role of depression in the progression of tumor cell inflammation and the potential regulatory mechanisms of aerobic exercise: a narrative review from molecular and cellular perspectives

**DOI:** 10.3389/fpsyt.2025.1683058

**Published:** 2025-12-17

**Authors:** Mingming Ren, Jianda Kong, Chuankai Luan, Yujing Mu

**Affiliations:** 1 College of Sports Science, Qufu Normal University, Jining, China; 2 Beijing University of Traditional Chinese Medicine Dongzhimen Hospital, Beijing, China

**Keywords:** depression, tumor progression, inflammatory response, aerobic exercise, immune regulation, anti-tumor therapy

## Abstract

There is a tight correlation between depression, and tumor progression, particularly via the regulation of the immune system, and inflammatory responses. Chronic inflammation is a member of the core causes in the tumor microenvironment, which can promote tumor initiation, progression, and immune evasion. An increasing body of literature has reported that aerobic exercise (AE), as a non-pharmacological intervention, can display potential in anti-tumor therapy by modulating the immune system, delaying chronic inflammation, and increasing neurotransmitter balance. However, it is worth noting that extreme AE may cause negative influences, such as immunosuppression, which influences its anti-tumor efficacy. Our review aims to investigate how depression influences the inflammatory progression of tumor cells via immune regulation, and the potential regulatory processes of AE in this mechanism. Moreover, we further explore the potential of AE in tumor treatment, and delves into its potential deleterious impacts. via this literature review, together with perspectives from molecular, and cellular biology, notably, our review explores the influences of depression, and AE on the tumor microenvironment, and immune responses. It centers on the contribution of AE in modulating immune cell functions, delaying chronic inflammatory responses, and increasing neurotransmitter balance. Depression promotes inflammatory responses in the tumor microenvironment via neurotransmitter imbalance, abnormal activation of the hypothalamic-pituitary-adrenal axis, and immune system dysregulation, hence triggering tumor growth, and metastasis. AE can positively modulate the immune system, decrease inflammation, as well as improve tumor immune surveillance function. Moderate AE modulates immune responses in the tumor microenvironment in the context of enhancing the activity of immune cells, lowering the levels of pro-inflammatory factors, and improving the production of anti-inflammatory factors, hence blocking the growth, and spread of tumor cells. However, extreme AE may cause immunosuppression, influencing anti-tumor influences, so individualized changes to the intensity, and frequency of exercise interventions are needed.

## 1 Introduction

Depression is a prevalent neuropsychiatric disorder with systemic implications that extend beyond affective symptoms to influence various physiological processes through biological pathways such as neurotransmitter imbalance, hypothalamic-pituitary-adrenal (HPA) axis dysregulation, and immune dysfunction (1, 2). A growing body of evidence underscores a significant correlation between depression and accelerated tumor progression (3). This link is largely mediated by chronic inflammation, a hallmark of the tumor microenvironment that facilitates tumor initiation, progression, and immune evasion (4, 5). The biological underpinnings of depression—including dysregulated neurotransmitter systems, a hyperactive HPA axis, and elevated pro-inflammatory cytokines—collectively foster a systemic and local milieu conducive to tumor growth and metastasis (6). It is worth noting that the relationship between depression and cancer is bidirectional and complex. Not only can depression promote tumor development and progression through neuroimmune endocrine disorders, but cancer diagnosis itself, its rigorous treatment regimen (such as chemotherapy and radiation therapy), and the accompanying psychological and social pressure can also induce systemic inflammatory responses, activate the HPA axis, and other shared mechanisms, leading to or exacerbating depressive symptoms. “ (7). This two-way interaction forms a vicious cycle, collectively leading to a shortened survival period and decreased quality of life for patients. However, a comprehensive review of the molecular and cellular mechanisms underlying this bidirectional relationship is currently insufficient.

Concurrently, moderate aerobic exercise (AE) has emerged as a potent non-pharmacological intervention with demonstrated benefits for both mental and physical health. AE is recognized for its ability to modulate the immune system, mitigate chronic inflammation, and restore neuroendocrine balance (8). Within the oncology context, these actions can enhance anti-tumor immune surveillance and indirectly counter tumor-promoting pathways associated with depression (9). However, the therapeutic potential of AE is not without nuance, as excessive exercise may lead to transient immunosuppression, highlighting the critical need for optimized and personalized exercise prescriptions.

Despite the established connections between depression and cancer, and the promising role of AE, several critical knowledge gaps remain. First, the precise molecular and cellular mechanisms by which depression-induced neuroimmune dysregulation orchestrates the inflammatory tumor microenvironment are not fully elucidated. Second, while AE is beneficial, a comprehensive synthesis of its dual role—directly modulating anti-tumor immunity and indirectly ameliorating depression-driven tumor promotion—is lacking. Finally, the translation of these complex interactions into clear, evidence-based guidelines for exercise oncology, particularly for patients with comorbid depression, remains an unmet challenge.

This review, therefore, aims to bridge these gaps by providing a narrative synthesis from molecular and cellular perspectives. We will critically examine how depression fuels tumorigenesis via inflammatory pathways and delineate the potential regulatory mechanisms of AE within this framework. By integrating current evidence, this review seeks to offer a theoretical foundation for developing targeted exercise interventions in cancer care and to identify pivotal directions for future research.

## 2 Methods

This is a narrative review, not a systematic review, although we employed a systematic literature search strategy to ensure comprehensive coverage of relevant studies. Unlike systematic reviews, which rely on pre-defined inclusion criteria and quantitative data synthesis, this narrative review aims to provide an overview and critical discussion of the current literature on depression, immune modulation, and aerobic exercise (AE) in the context of cancer.

We retrieved literature from major databases, including Web of Science, PubMed, Embase, and PsycINFO, using keywords such as “depression,” “aerobic exercise,” “tumor progression,” and “immune regulation.” The search included studies published from database inception up to February 2025. After applying inclusion and exclusion criteria, a total of X studies were included in this review.

## 3 Findings

### 3.1 The implication of depression on the inflammatory progression of tumor cells

**Figure 1:**
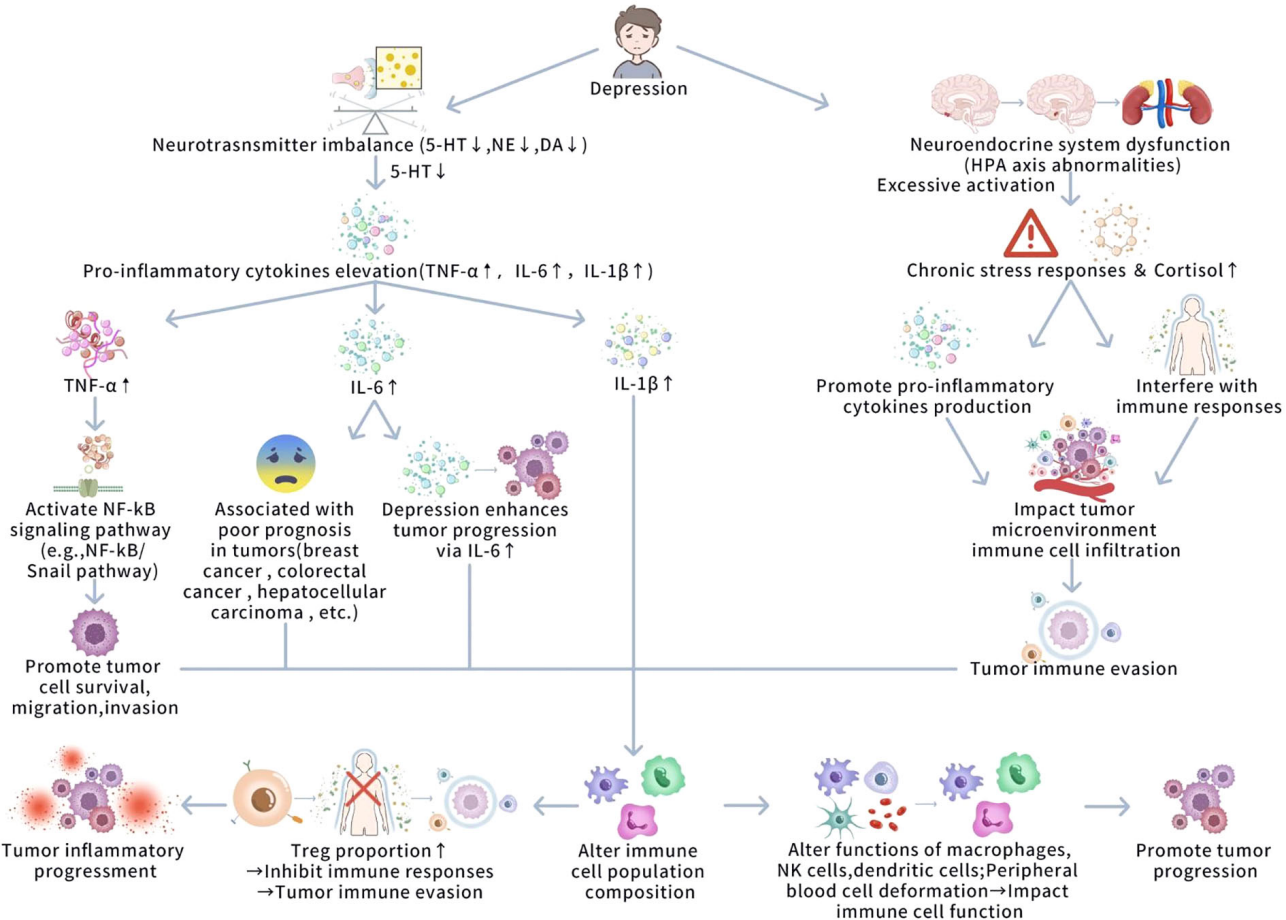
Schematic diagram of the mechanism by which depression regulates the tumor inflammatory microenvironment through the neuro immune endocrine axis. This figure illustrates how depression influences tumor inflammation through three main mechanisms: 1) neurotransmitter imbalance (decreased serotonin, norepinephrine, and dopamine); 2) overactivation of the HPA axis, leading to elevated cortisol levels; 3) increased secretion of pro-inflammatory cytokines (TNF-α, IL-6, IL-1β). These processes create a feedback loop that enhances tumor cell proliferation, migration, and invasion, increases immune suppressive cells (e.g., regulatory T cells), and impairs the anti-tumor function of immune cells like NK cells, macrophages, and dendritic cells. The dashed box highlights potential therapeutic targets, including neurotransmitter modulators, HPA axis inhibitors, and cytokine neutralizing antibodies.

Depression significantly reshapes the tumor microenvironment and promotes inflammation progression through neurotransmitter imbalance, excessive activation of the (Hypothalamic-Pituitary-Adrenal) HPA axis, and increased secretion of pro-inflammatory cytokines. However, the causal relationship between depression related inflammation and tumor immune escape remains controversial, especially the heterogeneous regulatory mechanisms in different tumor types are not yet clear. Next, we will explore in depth the new mechanisms by which depression affects tumor immune surveillance and its clinical translation prospects from the perspectives of immune checkpoint molecule expression, metabolic reprogramming, and neurotransmitter receptor targeted therapy. **Figure 1** illustrates the mechanism by which depression regulates the tumor inflammatory microenvironment through the neuroimmune endocrine axis.

#### 3.1.1 Biological processes of depression: a tripartite perspective

Depression is a complex neuropsychiatric disorder characterized by interrelated dysregulations across neurotransmitter systems, neuroendocrine function, and immune responses (10). These interconnected biological pathways collectively contribute to its pathophysiology and may also influence tumor-related processes.

##### 3.1.1.1 Neurotransmitter system dysregulation

A core pathophysiological feature of depression is the imbalance of key monoamine neurotransmitters. Deficiencies in serotonin (5-HT), norepinephrine (NE), and dopamine (DA) are strongly implicated in the onset and maintenance of depressive symptoms, directly affecting mood, motivation, and cognition (11).

##### 3.1.1.2 Neuroendocrine dysfunction: HPA axis hyperactivity

Dysregulation of the hypothalamic-pituitary-adrenal (HPA) axis is central to depression, with abnormal activity considered fundamental to its pathogenesis (12, 13). Chronic HPA axis activation results in sustained stress responses and elevated cortisol levels. This hypercortisolemia not only serves as a key biological marker of depression but also drives immune dysregulation and promotes a pro-inflammatory state (14, 15). Cortisol can further alter immune cell activity and infiltration within the tumor microenvironment, potentially facilitating tumor immune evasion (4).

##### 3.1.1.3 Immune-inflammatory activation and clinical correlates

Inflammatory processes are now recognized as a pivotal component of depression. This state is characterized by elevated levels of pro-inflammatory cytokines, including tumor necrosis factor-α (TNF-α) and interleukin-6 (IL-6) (16). Notably, reduced 5-HT levels have been linked to increased TNF-α and IL-6, creating a feed-forward loop between neurotransmitter imbalance and inflammation that may foster a microenvironment conducive to tumor growth and metastasis (16, 17). The clinical complexity of these biological mechanisms is exemplified by the challenge of treating treatment-resistant depression (TRD). A recent Delphi consensus underscores the need for tailored augmentation strategies, highlighting the heterogeneity in management and the emerging role of therapies like esketamine in refractory cases (15).

#### 3.1.2 Depression-induced remodeling of the tumor immune microenvironment

The immune system is a pivotal component of the tumor microenvironment (TME), critically influencing tumor progression and metastasis through immune cell activity, cytokine secretion, and dynamic crosstalk with tumor and stromal cells (4). Depression can create a favorable TME for tumor growth and metastasis primarily by dysregulating immune function and promoting a state of chronic inflammation.

##### 3.1.2.1 Core mechanism: elevation of pro-inflammatory cytokines

A hallmark of depression is a heightened level of pro-inflammatory cytokines, including Tumor Necrosis Factor-α (TNF-α), Interleukin-6 (IL-6), and Interleukin-1β (IL-1β) (18, 19). This inflammatory state is not merely a biomarker of depression but is mechanistically linked to tumor progression. Chronic inflammation can suppress normal immune cell function and provide a fertile ground for tumor cell survival, proliferation, and immune evasion (4, 19). For example, TNF-α promotes tumor cell survival, migration, and invasion, notably by activating the NF-κB signaling pathway and its downstream effectors like Snail (20, 21). Furthermore, IL-6 is a key immunoregulatory factor associated with poor prognosis in cancers such as breast and colorectal cancer (22). A positive correlation exists between elevated IL-6 levels and depressive symptoms, suggesting depression may accelerate tumor progression in part through enhancing IL-6 signaling (23, 24).

##### 3.1.2.2 Secondary mechanism: alteration of immune cell composition and function

Beyond cytokines, depression reshapes the TME by altering the population and function of key immune cells. There is typically an increased proportion of immunosuppressive regulatory T cells (Tregs) in depressed patients, which facilitate tumor immune evasion by inhibiting anti-tumor immune responses (25). Depression can also impair the function of other immune effector cells, including macrophages, natural killer (NK) cells, and dendritic cells. For instance, increased deformability of peripheral blood cells in depressed patients may hinder their immune function, further promoting tumor progression (6).

##### 3.1.2.3 Molecular mechanisms of the anti-inflammatory influence of aerobic exercise

Aerobic exercise (AE) counteracts the pro-tumorigenic TME through potent anti-inflammatory mechanisms, serving as a key process in its role in cancer treatment. AE directly modulates the inflammatory milieu by reducing the levels of key pro-inflammatory cytokines such as TNF-α, IL-6, and CRP, as evidenced in breast cancer studies (26, 27). Concurrently, AE can enhance the production of anti-inflammatory cytokines like IL-10 (28). A central molecular mechanism involves the suppression of the NF-κB signaling pathway, leading to reduced expression of TNF-α and IL-6, thereby lowering inflammation in the TME and inhibiting tumor growth and metastasis (29). In addition, chronic inflammation is closely linked to increased oxidative stress, which promotes tumorigenesis. AE mitigates this by boosting the activity of antioxidant enzymes, such as superoxide dismutase (SOD). This reduction in oxidative stress decreases inflammation-related gene expression and helps block tumor cell proliferation and invasion (30, 31). Most evidence derives from specific cancers like breast cancer, underscoring the need for cross-cancer validation. The effects of AE are highly dependent on its intensity, frequency, and duration. Critically, while moderate AE is beneficial, extreme AE may trigger inflammatory responses and transient “exercise-induced immunosuppression,” potentially hindering immune function and anti-tumor efficacy (32). Future research must therefore define optimal, individualized AE regimens to maximize anti-tumor immune responses while avoiding negative impacts.

##### 3.1.1.4 Pathophysiological mechanisms shared with cancer

It is worth emphasizing that the core biological changes of depression mentioned above, including neurotransmitter imbalance, HPA axis hyperactivity, and chronic inflammatory states, are not unique to depression. More and more evidence suggests that these are key pathophysiological mechanisms shared by depression and cancer (33). In cancer patients, the tumor itself, treatment side effects, and psychological stress can all serve as chronic stressors, continuously activating the innate immune system and HPA axis, leading to elevated levels of pro-inflammatory cytokines (such as TNF - α, IL-6) and cortisol (34, 35). These factors can directly induce depressive like behavior by affecting tryptophan metabolism and reducing levels of neurotrophic factors (34, 35). Therefore, mechanisms such as inflammation form the core biological bridge connecting cancer and depression, explaining why the two co-occur so frequently.

### 3.2 Regulatory role of AE in the inflammatory progression of tumor cells

#### 3.2.1 Regulation of the immune system by AE

Aerobic exercise (AE) has been reported to positively modulate the immune system, promote immune cell function, decrease chronic inflammation, and inhibit tumor progression. Literature has reported that moderate AE can promote the activity of the immune system, particularly by enhancing the quantity and function of immune cells (such as T cells, natural killer cells (NK cells), macrophages, etc.), and improve the body’s immune surveillance capacity, hence blocking the growth and spread of tumor cells (36, 37). The regulatory impact of AE is quantitatively supported by meta-analyses. For instance, one comprehensive meta-analysis concluded that exercise training significantly reduces pro-inflammatory markers in cancer survivors (Standardized Mean Difference, SMD: -0.2), with combined aerobic and resistance training showing the greatest effect (SMD: -0.3) (38).

The regulation of the immune system by AE is primarily demonstrated in the following aspects. It is worth noting that AE can vitally improve the function of immune cells and enhance anti-tumor immune responses. For instance, an article reported that AE can activate CD8+ T cells, decrease tumor growth, and this process is dependent on the contribution of CD8+ T cells (39). Moreover, AE can also enhance the number of NK cells and improve their direct killing impact on tumor cells (40). A meta-analysis focusing on breast cancer patients and survivors further substantiates that exercise interventions do not negatively affect the number or activity of key anti-tumor immune cells, indicating that exercise prescription is safe from an immunological perspective and should not be discouraged (41). These findings collectively determine that AE plays a vital role in enhancing immune cell function and improving tumor immune surveillance.

Besides, AE can decrease the number of inhibitory immune cells, such as regulatory T cells (Tregs), which are central to the mechanism of tumor immune evasion (42). By blocking the function of Tregs, AE helps improve the body’s anti-tumor immune response. Literature has reported that endurance training can inhibit the recruitment of FoxP3+ Tregs cells in tumors, hence enhancing anti-tumor immunity (43). Moreover, it is interesting that AE training can also promote tumor immune control by enhancing the infiltration of CD8+ T cells via the CXCR3 signaling pathway, hence rendering breast cancer more sensitive to immune checkpoint inhibition therapy (44). Furthermore, AE promotes the polarization of macrophages towards the M1 type (pro-inflammatory type) by modulating their polarity, hence enhancing the efficacy of tumor immune surveillance. This transformation promotes the phagocytic impact of macrophages on tumor cells, further delaying tumor progression. An article reported that AE can promote the polarization of macrophages concerning the M1 type by modulating their polarity, hence enhancing the efficacy of tumor immune surveillance (45).

**Figure 2:**
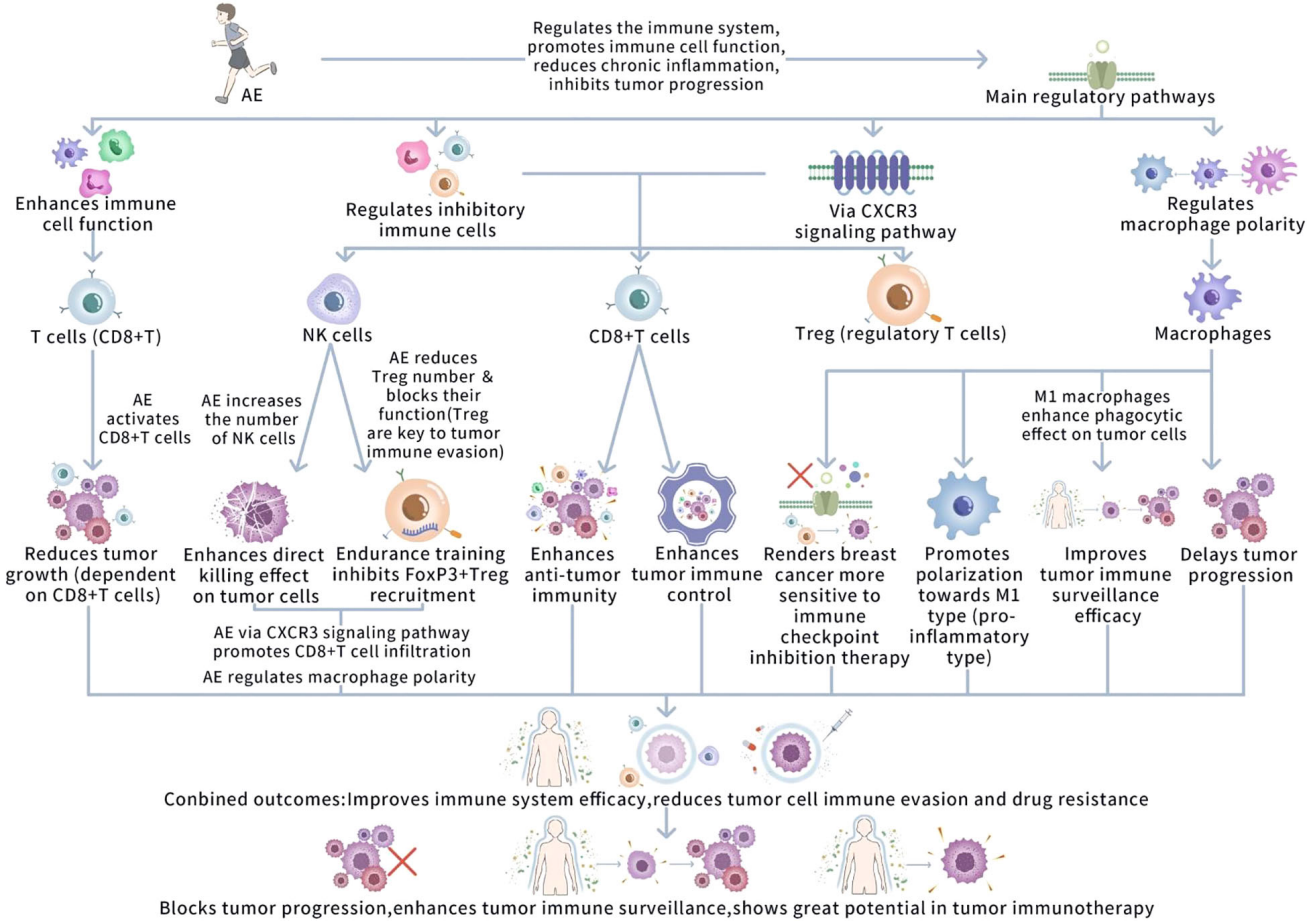
Immune system regulation in tumor monitoring and progression mediated by AE. This figure outlines how AE enhances anti-tumor immunity through several mechanisms: 1) activation of CD8+ T cells and NK cells, leading to cytotoxic activity against tumors; 2) reduction of regulatory Tregs, which are key to tumor immune escape; 3) promotion of CD8+ T cell infiltration into tumors via the CXCR3 signaling pathway, sensitizing tumors to immune checkpoint inhibitors; and 4) macrophage polarization towards the M1 (pro-inflammatory) phenotype, enhancing tumor cell phagocytosis. These processes improve immune surveillance, reduce tumor immune evasion, and inhibit cancer progression, positioning AE as a potential adjunct in tumor immunotherapy.

Through these processes, in conclusion, AE can vitally improve the efficacy of the immune system, decrease the immune evasion and drug resistance of tumor cells, hence positively blocking tumor progression. AE not only promotes the tumor immune surveillance ability by enhancing the function of immune cells but also exerts influences via multiple aspects, such as modulating the function of inhibitory immune cells like Tregs and the polarity of macrophages, demonstrating its great potential in tumor immunotherapy. **Figure 2** illustrates immune system regulation in tumor monitoring and progression mediated by AE.

#### 3.2.2 Molecular processes of anti-inflammatory influences

The anti-inflammatory impact of AE is one of the key mechanisms by which it plays a vital role in cancer treatment. Literature has displayed that moderate AE can decrease the levels of pro-inflammatory factors via various molecular processes, decrease chronic inflammatory responses in the tumor microenvironment, and hence inhibit the growth and metastasis of tumor cells (46). Meta-analytic evidence substantiates this effect; for instance, a recent systematic review and meta-analysis focusing on overweight and obese women with breast cancer demonstrated that exercise significantly reduced levels of C-reactive protein (CRP) (MD: -0.52, 95% CI: -0.94 to -0.11) and interleukin-6 (IL-6) (MD: -0.87, 95% CI: -1.62 to -0.11), although the reduction in tumor necrosis factor-α (TNF-α) was not statistically significant (47).

It is worth noting that AE can decrease pro-inflammatory cytokines in the tumor microenvironment, such as TNF-α, IL-6, and CRP, by modulating immune system responses, and these cytokines play a promoting role in tumor progression. Literature has reported that higher-intensity AE is linked to lowered levels of CRP, IL-6, and TNF-α, and this has been determined in breast cancer (26). Besides, AE can also delay chronic inflammatory responses by enhancing the levels of anti-inflammatory cytokines, such as IL-10. It is worth noting that AE is considered to positively modulate the inflammatory state in the body, decrease the levels of pro-inflammatory factors, as well as improve the production of anti-inflammatory factors, such as IL-10 (28). The potential role of these immune response regulations in anti-cancer therapy cannot be ignored. By modulating the NF-κB pathway, AE can inhibit the expression of pro-inflammatory factors TNF-α and IL-6, hence lowering the inflammatory response in the tumor microenvironment and blocking tumor growth and metastasis (29). An article reported that the levels of IL-6 and TNF-α in the serum of breast cancer patients lowered vitally after AE, determining that AE can positively modulate the levels of these pro-inflammatory factors (27). Besides, another study noted that AE therapy inhibits tumor growth by delaying chronic inflammatory responses related to cancer, determining that AE may be a positive means to delay cancer progression (48).

Long-term chronic inflammation can cause increased oxidative stress, which is tightly linked to tumorigenesis. It is worth noting that AE can decrease oxidative stress by enhancing the activity of antioxidant enzymes (such as superoxide dismutase, SOD), hence decreasing inflammation-related gene expression and blocking the proliferation and invasion of tumor cells. For instance, dendritic cells (DCs) display high-level expression of the antioxidant enzyme manganese superoxide dismutase (Mn-SOD) during their differentiation and maturation (30). Besides, macrophages respond to oxidative stress by enhancing glutathione synthesis and mitochondrial function when exposed to copper-based nanoparticles. This molecular response may have vital implications for the progression of atherosclerosis (31). Through these processes, AE can positively delay chronic inflammation in the tumor microenvironment, hence blocking the malignant progression of tumors and offering a potential new approach for anti-tumor therapy. However, these articles primarily center on specific cancer types (such as breast cancer) and lack cross-cancer validation. The implication of the intensity, frequency, and duration of AE on the immune system varies from person to person; extreme AE may trigger inflammatory responses and hinder immune function. Besides, extreme AE may cause “AE-induced immunosuppression”, particularly significant extreme AE (32). Further studies should further investigate the appropriate intensity and frequency of AE to indicate that it promotes anti-tumor immune responses without causing negative influences.

**Figure 3:**
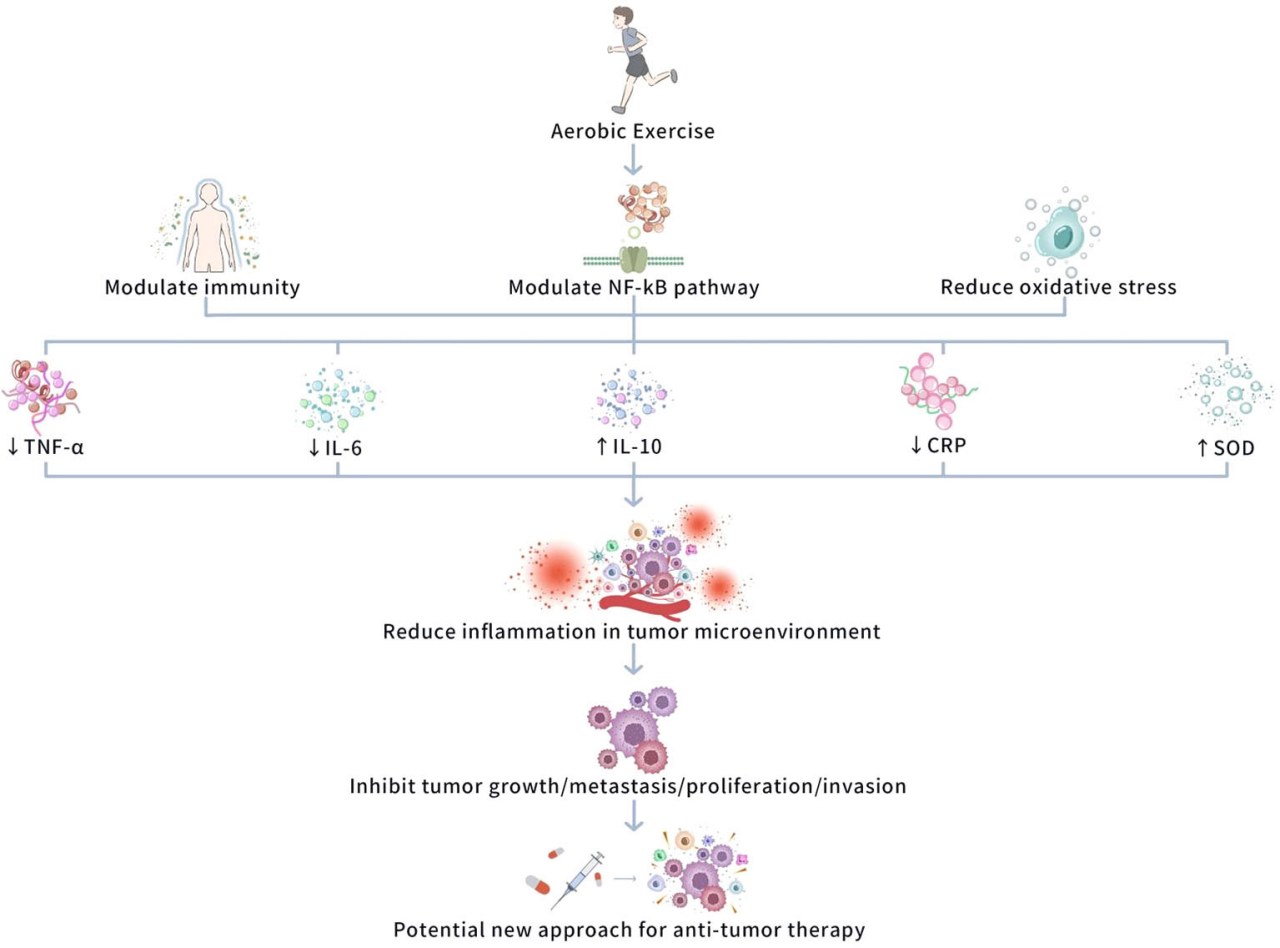
Schematic diagram of the molecular mechanism of AE anti-inflammatory effect and its anti-tumor effect. This figure illustrates how AE exerts anti-inflammatory effects through immune regulation, NF-κB pathway inhibition, and oxidative stress modulation, thereby inhibiting tumor growth, metastasis, proliferation, and invasion. AE reduces pro-inflammatory factors (e.g., TNF-α, IL-6, CRP) and increases anti-inflammatory factors (e.g., IL-10), alleviating chronic inflammation in the tumor microenvironment. AE also inhibits the NF-κB pathway, reducing pro-inflammatory cytokines, and enhances antioxidant enzyme activity (e.g., SOD, Mn-SOD) to reduce oxidative stress. These combined effects inhibit tumor progression, offering potential targets for anti-tumor therapy.

Overall, AE has potential therapeutic value in tumor treatment via its anti-inflammatory influences, but its specific efficacy depends on multiple factors, comprising the type and intensity of AE and the individual’s physiological state. Therefore, how to precisely adjust AE intervention approaches in clinical practice to achieve optimal anti-inflammatory and anti-tumor influences remains a vital direction for future literature. **Figure 3** shows a schematic diagram of the molecular mechanism of AE anti-inflammatory effect and its anti-tumor effect.

#### 3.2.3 The impact of AE on delaying depressive symptoms

The regulatory impact of AE on neurotransmitters, particularly in the context of enhancing the production of 5-hydroxytryptamine (5-HT), norepinephrine, and dopamine, has been reported to positively improve depressive symptoms. The antidepressant effect of AE is strongly supported by meta-analytic evidence. For instance, a meta-analysis of randomized controlled trials in older adults found that moderate-to-vigorous physical exercise intervention was associated with a medium effect size (Hedges’ g = 0.64, 95% CI: 0.27 to 1.01) in reducing depressive symptoms (49). Furthermore, the benefits extend to specific patient populations; a meta-analysis on multiple sclerosis patients demonstrated that remote exercise and physiotherapy programs were significantly more effective than control interventions for managing depression (Hedges’ g = -0.41, 95% CI = -0.74 to -0.09) (50). Literature has reported that AE not only promotes mood by modulating neurotransmitter imbalance but also decreases immune responses caused by these imbalances, delays inflammatory reactions, and hence indirectly inhibits tumor growth and metastasis (37). It is worth noting that according to the literature of Mead et al., more importantly, physical exercise is considered a positive treatment for depression by the UK health authorities, and this conclusion comes from a systematic review of 23 articles (51).

The implication of AE on the endocrine system plays a vital role in the treatment of depression. Depression patients are usually accompanied by elevated cortisol levels, and excessive production of cortisol is tightly linked to the activation of chronic inflammation, hence promoting tumor progression (52). Literature has reported that there is a vital correlation between elevated cortisol levels and depression, and extreme cortisol production may cause the deterioration of emotional and physical health and even drive the progression of chronic diseases (53). On the other hand, AE can decrease cortisol levels by modulating the HPA axis, hence alleviating depressive symptoms. AE has been displayed to positively delay depressive symptoms, as well as improve the physical and psychological status of depression patients in the context of lowering cortisol production (54).

**Figure 4:**
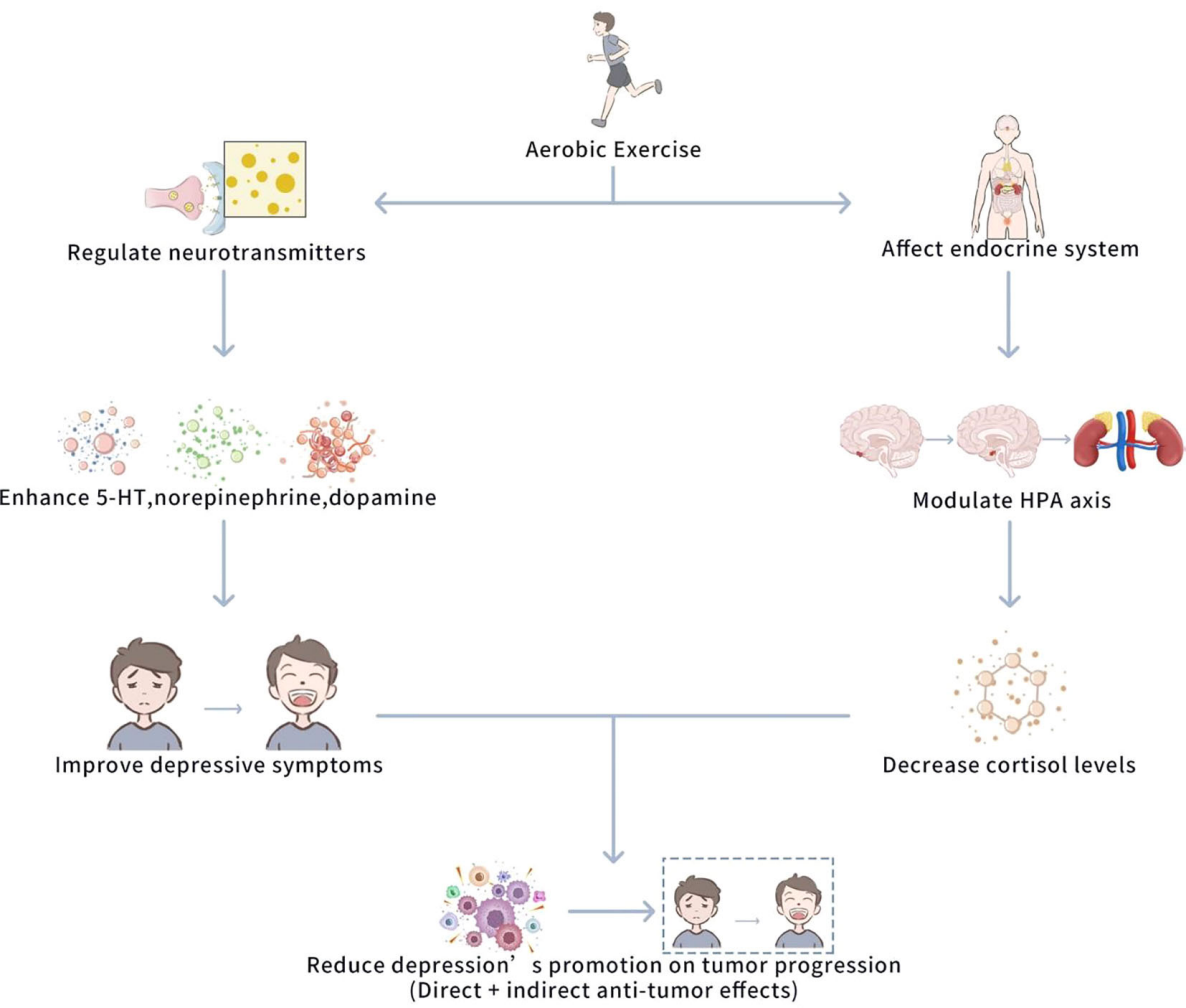
Schematic diagram of the mechanism of the effect of aerobic exercise on delaying depressive symptoms. This figure illustrates how AE delays depressive symptoms through two main mechanisms: 1) regulation of neurotransmitters (5-HT, norepinephrine, dopamine), and 2) modulation of the HPA axis to reduce cortisol levels. These actions together reduce depression-driven tumor progression and inhibit tumor growth and metastasis by alleviating immune and inflammatory responses linked to neurotransmitter imbalance.

Thus, AE not only exerts anti-tumor influences by directly modulating the immune system and inflammatory responses but also indirectly decreases the promoting impact of depression on tumor progression by alleviating depressive symptoms. Although the delaying impact of AE on depression has been positively investigated, there are vital individual differences in these influences. AE of various intensities and types may exert different influences on various individuals, and extreme AE may even cause negative influences. Further research should focus on determining the optimal AE regimen to ensure that it can positively delay depressive symptoms without causing negative influences. **Figure 4** shows a schematic diagram of the mechanism of the effect of aerobic exercise on delaying depressive symptoms.

#### 3.2.4 Potential negative influences of AE

Despite the established benefits of moderate aerobic exercise (AE) on immune function and inflammation, extreme or high-intensity AE can exert deleterious impacts on the immune system and overall health (55). Prolonged, high-intensity AE may induce a state of transient immunosuppression, often termed “exercise-induced immunosuppression,” which can temporarily compromise the body’s immune surveillance against tumors and pathogens (36, 56). This phenomenon is characterized by a temporary weakening of immune responses, particularly in the immediate post-exercise period (36). Furthermore, extreme exercise can elevate oxidative stress, which in turn inhibits immune cell function and paradoxically promotes inflammatory responses, potentially undermining anti-tumor efficacy and facilitating tumor immune escape (57).

Consequently, while moderate AE is beneficial, excessive exercise can lead to a decline in immune function. To maximize the anti-tumor benefits of AE while avoiding these adverse effects, future research must prioritize defining the optimal intensity, frequency, and duration of exercise, and develop personalized intervention programs tailored to individual health status.

#### 3.2.5 Optimization of exercise prescription: the influence of dose, intensity, and individual differences on tumor outcome

Although the mechanisms and clinical evidence described in this review establish an active role for aerobic exercise (AE) in oncology treatment, its ultimate effect is not static but highly dependent on the specific parameters of the exercise prescription, and it is important to understand the role of aerobic exercise in cancer therapy, including dose, intensity, frequency and individual differences in patients and compliance. A deep understanding of these variables is key to transforming AE from a generic health recommendation into a precision oncology treatment adjunct.

Exercise intensity is the core variable that determines the physiological effects of AE, and its relationship with tumor outcome often shows a”U-shaped” or”J-shaped” curve. A large amount of evidence shows that moderate intensity aerobic exercise has been widely proved to inhibit tumor progression through a variety of mechanisms. For example, a study in a mouse model of colon cancer found that moderate-intensity aerobic exercise training reduced tumor growth and improved survival. Its mechanism is related to improving tumor hypoxia, increasing infiltration of CD8 + tumor infiltrating lymphocyte (TIL), and enhancing CD8 + TIL effector function by increasing mitochondrial content and function (58). In humans, the anti-inflammatory properties of moderate-intensity AE have also been supported by systematic reviews showing that it significantly reduces levels of IL-6, c-reactive protein (CRP), and tumor necrosis factor (TNF-α), this is essential to Counteract The tumor-associated chronic inflammatory state (59). In addition, regular AE can also optimize the redox balance of the body by lowering oxidative stress markers (such as MDA) and increasing antioxidant capacity (such as SOD and TAC), this may indirectly inhibit the malignant progression of tumors (60).

Conversely, prolonged, high-intensity, and insufficiently recovered AES may induce acute oxidative stress and inflammation, resulting in temporary”Exercise-induced immunosuppression.”. This period, characterized by a temporary decrease in the number of circulating lymphocyte and impaired immune cell function, could theoretically provide a time window for tumor immune escape, thereby impairing the antitumor efficacy of AE. A systematic review noted that high-intensity exercise often leads to significant increases in higher levels of inflammatory mediators such as IL-6, as well as white blood cells and creatine kinase (CK), and this may be associated with increased risk of tissue damage and chronic inflammation (61). The immunosuppression experienced by athletes during prolonged high-intensity training also suggests the importance of balancing nutrition and training. Felice, F., et al. (2024). Is Micronutrient Supplementation Helpful in Supporting the Immune System during Prolonged, High-Intensity Training. This highlights the importance of avoiding extreme AES and ensuring full recovery.

The overall dose of exercise, which is often determined by a combination of frequency, duration and intensity, is also crucial. Observational studies and partial intervention trials have shown that a higher total amount of exercise is associated with a lower risk of cancer recurrence and cancer-specific mortality over a moderate intensity range. A systematic review and Meta-analysis of 136 studies found that, higher levels of total or leisure-time physical activity before and after diagnosis were associated with improved survival for at least 11 cancers, including breast, colorectal, and colorectal cancers, an inverse dose-response relationship between PA and breast cancer-specific and all-cause mortality was observed, risk drops precipitously at 10-15met-hours per week (62). Another dose-response Meta-analysis of domestic physical activity further supported the linear inverse association between physical activity and reduced cancer risk (63).

Regular exercise frequency (e.g. 3–5 times per week) is critical for maintaining a stable and beneficial immune and endocrine environment. A systematic review and Meta-analysis confirmed that regular moderate-to vigorous-intensity physical activity not only reduces the risk of community-acquired infectious diseases and infectious disease mortality, but also reduces the risk of infection, it also strengthens the immune system’s first line of defense (e.g., by increasing CD4 count and salivary IGA concentration), effects of Regular Physical Activity on the Immune System, Vaccination and Risk of Community-Acquired Infectious Disease in the General Population: Systematic Review and meta-analysis. Sports Medicine. This steady-state regulation of the systemic microenvironment is thought to influence hallmark features of Cancer possibly by reshaping the distal tumor microenvironment (64). On the contrary, the irregular movement pattern is difficult to produce a sustained positive remodeling of the tumor microenvironment.

However, perfect exercise prescription, if the patient cannot adhere to, its efficacy will be greatly reduced. Patients’ compliance directly determines the actual exercise”Dose” they receive. In oncologic patients, adherence is influenced by a combination of cancer type, treatment side effects (such as cancer-related fatigue), psychological status (such as depression, as described in section 2.3.1), and Social Support Systems. This means that there is no one-size-fits-all exercise plan. Different tumor types, such as breast cancer and pancreatic cancer, have very different immune microenvironments and may respond differently to the same exercise stimuli. For example, Pancreatic cancer is characterized by its highly immunosuppressive Microenvironment and resistance to Immunotherapies (65). Whereas in breast cancer models, studies have shown differences in CD4 + and CD8 + t-cell dynamics after immune checkpoint inhibitor therapy, even in immune”Cold” tumors, 10,10,11,11, this suggests that the pattern of immune response in different tumor types may influence the ultimate effect of exercise intervention (66). The physical status and exercise tolerance of patients during adjuvant therapy and advanced palliative care vary greatly; at the same time, chemotherapy, radiotherapy, or immunotherapy may change the physiological response of patients to exercise. The systematic review confirmed that exercise intervention is safe and feasible in the palliative care phase of advanced cancer, and that it can improve the outcome of patients with advanced cancer, the effects of physical exercise in the palliative care phase for people with advanced cancer: A systematic review with meta-analysis. Journal of cancer survivorship. In addition, the patient’s age, physical ability, comorbidities and personal preferences must be taken into account in the formulation of exercise prescriptions. The international multidisciplinary roundtable consensus emphasizes that cancer survivors should be provided with exercise prescriptions that target specific cancer types, treatment options, and/or health outcomes, and recommends that each survivor should”Avoid inactivity,” and that all survivors should”Avoid inactivity.” At the same time, specific doses of aerobic, resistance, or combined exercise are recommended based on available evidence to improve functional outcomes such as anxiety, depression, and fatigue (67).

In summary, the effects of AE on tumors are regulated by a complex system involving intensity, dose, frequency, and compliance. Future research directions should aim to clarify the dose-effect relationship between different exercise parameters and specific tumor outcomes through well-designed clinical trials, and to develop truly personalized, dynamically adjusted exercise prescriptions, as well as to improve the clinical outcomes of cancer patients, to maximize the therapeutic potential of AE in psycho-oncology.

#### 3.2.6 Comparison of exercise modes and synergistic effects of diverse lifestyles

##### 3.2.6.1 Comparison and combination of aerobic exercise and resistance training: potential for synergistic enhancement

Different exercise modes affect tumor progression through unique and complementary physiological mechanisms. The core advantages of AE lie in improving cardiopulmonary function, enhancing systemic energy metabolism, and effectively regulating systemic inflammation and immune function, such as promoting the infiltration of natural killer (NK) cells and CD8+T cells into tumor tissue, thereby affecting the tumor microenvironment (TME) (68).

In contrast, the core benefit of Resistance Training (RT) is to increase and maintain lean body mass (muscle mass). This is crucial in oncology, as muscle loss caused by cancer cachexia is directly associated with poorer treatment tolerance, higher risk of functional impairment, and shorter survival (69). Cancer cachexia is caused by systemic inflammation upregulation and increased catabolic stimulation, leading to inhibition of protein synthesis and enhanced muscle breakdown (70). In addition, RT may independently generate signals that inhibit tumor growth by activating and releasing specific myogenic factors, such as irisin. Research has shown that irisin can induce G2/M phase arrest in glioblastoma cells, increase p21 levels, and inhibit cell proliferation and invasion (71). A systematic review and meta-analysis further suggest that irisin is downregulated in various cancers and may play an important role in tumor progression and metastasis by participating in multiple signaling pathways (72). Clinical evidence on efficacy comparison is constantly accumulating. A comprehensive meta-analysis covering cancer survivors found that although AE and RT can each bring benefits, combined training (CT) - which integrates aerobic and resistance elements in the same intervention regimen - showed the greatest effect value in reducing key pro-inflammatory markers such as C-reactive protein (38). This suggests that compared with single mode exercise, CT may produce more powerful anti-inflammatory effects by simultaneously targeting multiple physiological systems (immunity, metabolism, muscle).

Therefore, based on the complementarity of its mechanism and preliminary evidence of synergy, for most cancer survivors, the joint program containing aerobic and resistance training is likely to bring more comprehensive physiological benefits than any single model, and is an ideal exercise strategy to achieve “synergy” and optimize long-term prognosis.

##### 3.2.6.2 Beyond exercise: integration of key lifestyle variables

Maximizing the benefits of AE often requires placing it within a broader ecosystem of healthy behaviors. Other lifestyle variables such as nutrition, sleep, and stress management interact with exercise to shape the internal environment of the body, which in turn affects the occurrence and development of tumors.

A healthy dietary pattern, such as the Mediterranean diet, is rich in anti-inflammatory and antioxidant substances, which have a protective effect on various chronic diseases, including cancer (73). When combined with exercise, proper nutrition provides a crucial material foundation for post exercise physical recovery, maintenance of muscle mass, and optimization of the immune system. Research has shown that mixed intake of flavonoids, n-3 fatty acids, and vitamin C can effectively reduce the increase of oxidative stress markers after intense exercise, and this effect is independent of changes in plasma antioxidant capacity (74). A systematic review and meta-analysis further confirmed that the combination of physical activity and diet can create a synergistic effect in cancer patients, jointly creating a metabolic inflammatory environment that is unfavorable for tumor growth (75).

Sleep disorders are closely related to a series of physiological changes that promote tumor progression. Sleep, especially deep sleep, has inhibitory effects on the HPA axis, and activation of the HPA axis or administration of glucocorticoids can lead to wakefulness and insomnia. Insomnia is associated with a 24-hour increase in ACTH and cortisol secretion. In addition, pro-inflammatory cytokines IL-6 and TNF-α are elevated in diseases associated with excessive daytime sleepiness, and sleep deprivation can lead to excessive daytime secretion of sleepiness and IL-6 (76). Regular AE has been confirmed by multiple studies as a non-pharmacological approach to improve sleep quality and efficiency in cancer patients (77). Therefore, improving sleep through exercise can indirectly reduce systemic inflammation, enhance immune surveillance, and thus form a positive cycle with the direct anti-tumor effect of AE.

Chronic psychological stress is a key factor driving neuroendocrine disorders and inflammation. Combining AE with structured stress management techniques such as mindfulness, meditation, or cognitive-behavioral therapy can achieve multi-target regulation of the HPA axis and sympathetic nervous system. For example, a study on non-small cell lung cancer patients showed that a combined intervention of exercise and mindfulness based stress reduction (MBSR) showed superior effects than conventional care in improving patient anxiety, depression, and sleep quality (78). This proves that integrating physical and mental interventions with physical activity may provide a more comprehensive solution for alleviating psychological distress and its downstream physiological hazards.

#### 3.2.6 Integrating AE into psycho-oncology care

##### 3.2.6.1 Improving mental health through neurobiological mechanisms

Cancer patients frequently experience high levels of psychological distress, including symptoms of depression and anxiety, which can severely impact their quality of life. Depression, in particular, is a common comorbidity that can exacerbate immune dysfunction and complicate treatment outcomes (79). The neurobiological underpinnings of this distress involve dysregulation of neurotransmitters and the hypothalamic-pituitary-adrenal (HPA) axis. Fortunately, AE has been demonstrated to alleviate depressive symptoms by modulating these very systems. A comprehensive systematic review concluded that exercise exerts its antidepressant effects through the regulation of HPA axis activity (e.g., reducing cortisol levels) and the enhancement of neurogenesis (e.g., increasing Brain-Derived Neurotrophic Factor (BDNF)) (80). By improving mood and emotional stability, AE can help restore the motivation and energy that are often diminished in depressed patients.

##### 3.2.6.2 Enhancing immune function and anticancer immunity

Beyond its psychological benefits, AE plays a crucial role in modulating immune function, which is vital in the context of cancer. Regular physical activity is associated with lower cancer incidence, mortality, and recurrence rates (81). This antitumorigenic effect is largely attributed to improved cancer immunosurveillance. AE promotes the activity and tumor infiltration of key immune cells, such as CD8+ T cells and natural killer (NK) cells, thereby enhancing the body’s ability to combat cancer progression (82). Within the framework of psycho-oncology care, integrating AE not only addresses emotional well-being but also directly bolsters the body’s physiological defenses against cancer, offering a holistic approach to patient care.

##### 3.2.6.3 Reducing inflammation and remodeling the tumor microenvironment

Conventional cancer treatments, such as chemotherapy and radiotherapy, often induce systemic inflammation and immune suppression, creating a microenvironment conducive to tumor growth and metastasis (83). Chronic inflammation is now recognized as a hallmark of cancer, playing a key role in its development, progression, and resistance to therapy (84). AE can counteract this by modulating cytokine levels and restoring the balance between pro-inflammatory and anti-inflammatory factors. Specifically, exercise has been shown to beneficially modulate the expression of chemokines—key signaling proteins in the tumor microenvironment (TME). By reducing pro-inflammatory chemokines and enhancing the recruitment of antitumor immune cells, AE helps create a TME less favorable to tumor growth (85). This anti-inflammatory effect is particularly significant in psycho-oncology, as reducing inflammation can help mitigate the side effects of cancer treatments and improve overall patient outcomes.

##### 3.2.6.4 The imperative for personalized exercise interventions

Given the diversity in patients’ physical conditions, treatment status, and psychological needs, personalized exercise interventions are essential to maximize the benefits of AE in psycho-oncology. Tailoring exercise programs to an individual’s capacity and preferences is critical for optimizing both psychological and immunological outcomes. For instance, moderate-intensity AE (e.g., brisk walking, cycling) has been shown to be highly effective in reducing depressive symptoms (86) and is generally considered beneficial for immune function without inducing the immunosuppressive effects sometimes seen with prolonged, high-intensity exercise (87). Therefore, integrating AE into standard psycho-oncology care requires a personalized approach that carefully considers the patient’s overall health status, preferences, and treatment plan.

##### 3.2.6.5 Implementation in clinical settings and multimodal integration

While the integration of AE into psycho-oncology care faces challenges related to infrastructure and support, its importance is increasingly recognized. Many cancer centers are now incorporating structured exercise programs. However, a systematic review of implementation outcomes highlighted that while feasibility and adoption are commonly reported, metrics like long-term sustainability and penetration into standard care are critically under-evaluated (88). Establishing exercise as a universal component of oncology requires multidisciplinary collaboration among oncologists, physiatrists, physical therapists, and exercise professionals to create effective referral pathways and community-based programs (89).

Furthermore, AE can be effectively combined with other psycho-oncological modalities, such as cognitive-behavioral therapy or mindfulness-based stress reduction (MBSR), to provide comprehensive support. For example, a study on patients with non-small cell lung cancer demonstrated that a combined intervention of MBSR and exercise led to significantly greater improvements in anxiety, depression, and sleep quality compared to conventional care alone (78). Similarly, evidence supports the efficacy of such combined mind-body and physical activity interventions for improving mood and quality of life even in challenging populations like brain tumor patients (90).

##### 3.2.6.6 Evidence for AE intervention in depression cancer comorbidity patients: from potential to practice

Although specialized AE intervention studies for cancer patients diagnosed with severe depression are still limited, in recent years, large-scale high-quality evidence targeting cancer patient populations (including a large number of individuals with clinically significant depressive symptoms) has provided strong support for the clinical application of AE in managing depression cancer comorbidities.

An authoritative systematic review and meta-analysis published in JAMA Network Open provides the most direct evidence for this. This study included 25 randomized controlled trials (RCTs) involving a total of 1931 adult cancer patients. The analysis results showed that compared with the control group (conventional care, waiting list, or attention control), aerobic physical activity (APA) was significantly associated with a decrease in self-reported depression severity in the short term (within 1 month after intervention) (standardized mean difference SMD=-0.38; 95% CI, -0.59 to -0.18; P <.001) (91). More importantly, this positive effect was maintained during long-term follow-up (6 to 12 months after the intervention) (SMD=-0.32; 95% CI, -0.60 to -0.04; P = .03). This finding suggests that the benefits of AE on depressive symptoms in cancer patients are not temporary effects but have sustained positive effects (91). This conclusion has been further validated in a specific vulnerable subgroup - elderly cancer patients. A meta-analysis of cancer patients aged 60 and above (including 27 RCTs, n=1929) also found a significant correlation between exercise intervention and a significant reduction in depression levels (SMD=-0.53; 95% CI, -0.79 to -0.28) and anxiety levels (SMD=-0.39; 95% CI, -0.66 to -0.12). The study also suggests that physical and mental exercises such as Tai Chi and yoga may show a stronger association in improving depression and anxiety (92). These pieces of evidence collectively indicate that AE is an effective non pharmacological means of improving the mental health of cancer patients, including elderly patients. Observational studies reveal the close relationship between lifestyle and psychological state from another perspective. A survey based on a nationally representative sample in the United States found that frequent non-medical use of marijuana is significantly associated with a higher risk of depression among cancer survivors, suggesting that certain coping strategies may not be beneficial or even harmful to mental health (93). In contrast, AE, as a positive and healthy behavioral intervention, demonstrates particularly valuable antidepressant benefits.

Based on the latest clinical evidence, we can draw a clear conclusion: although more forward-looking RCTs specifically designed for depression cancer comorbidities are needed in the future to optimize prescriptions, existing data has fully demonstrated that AE is an effective, persistent, and safe strategy associated with reducing depressive symptoms in cancer patients. This provides a solid and convincing evidence-based medicine foundation for translating the molecular mechanism mentioned in this article, namely AE exerting antidepressant and anti-tumor effects by regulating neurotransmitters, HPA axis function, and systemic inflammation levels, into specific clinical practice.

### 3.3 Interaction between depression, and the regulatory mechanism of AE

#### 3.3.1 The bidirectional interaction between depression and AE

**Figure 5:**
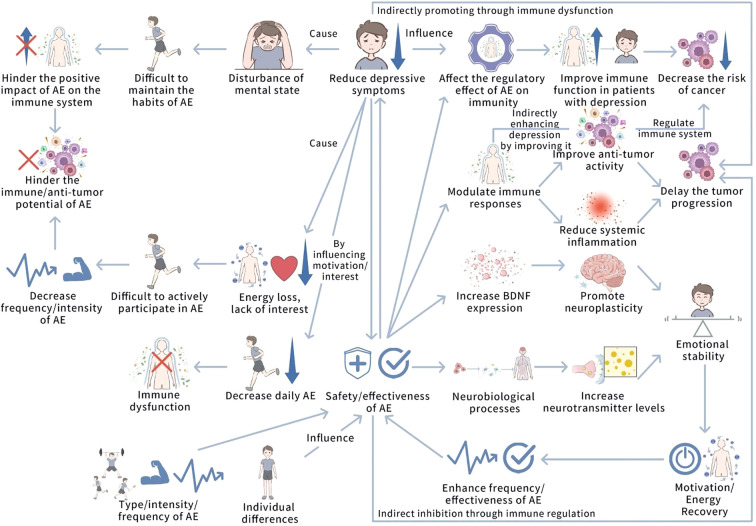
The interaction and interference relationship between depression and AE. This figure illustrates the bidirectional interaction between depression and AE. Depression impairs AE participation by reducing motivation and energy, weakening AE’s positive impact on immune function and anti-tumor potential, and potentially causing immune dysfunction. AE, by increasing neurotransmitters like BDNF, 5-HT, and norepinephrine, improves neural plasticity and cognitive functions, alleviating depressive symptoms and restoring motivation, creating a positive feedback loop. AE enhances anti-tumor immunity, regulates immune responses, and reduces inflammation, while depression may indirectly increase tumor risk through immune dysfunction. Individual differences and AE intensity can influence these interactions, potentially triggering negative effects.

The relationship between depression and aerobic exercise (AE) is bidirectional and dynamic. While AE holds promise for alleviating depression and inhibiting tumor progression, this interaction is complex and influenced by several factors, including the severity of depression and the parameters of the exercise regimen. **Figure 5** shows the interaction and interference relationship between depression and AE.

##### 3.3.1.1 The inhibitory impact of depression on AE participation and efficacy

The core symptoms of depression, such as lack of pleasure, insufficient energy, and decreased motivation, pose significant obstacles for patients to initiate and persist in adverse events (1, 94). It should be noted that in cancer patients, this barrier is amplified in both directions. On the one hand, depression itself weakens motivation; On the other hand, cancer and its treatments (such as fatigue, pain, and immune suppression) directly lead to physiological motor limitations and emotional distress (95). The decrease in frequency and intensity of AE caused by the combined effects of cancer and depression directly weakens its potential to regulate the immune system and exert anti-tumor effects. Particularly, depression exerts a significant negative impact on an individual’s capacity to engage in AE. Core symptoms such as anergy, anhedonia, and diminished motivation create a substantial barrier to initiating and maintaining regular exercise (1, 94). This reduction in AE frequency and intensity directly undermines its potential to modulate the immune system and exert anti-tumor effects (94, 95). Consequently, the presence of depression can initiate a vicious cycle, wherein depressive symptoms limit exercise, which in turn prevents the experience of exercise-induced physiological and psychological benefits.

##### 3.3.1.2 The therapeutic potential of AE in breaking the cycle

Conversely, AE can function as a powerful tool to disrupt this cycle by directly alleviating depressive symptoms. A key neurobiological mechanism involves the exercise-mediated upregulation of Brain-Derived Neurotrophic Factor (BDNF) and key neurotransmitters. Evidence confirms that AE training significantly increases circulating BDNF levels, which is correlated with improvements in depressive symptoms (96, 97). By promoting neuroplasticity and stabilizing mood through the modulation of 5-HT, norepinephrine, and dopamine, AE effectively restores energy, motivation, and emotional stability (98, 99). This improvement in mental state subsequently enhances exercise capacity, adherence, and overall effectiveness, establishing a positive feedback loop.

##### 3.3.1.3 The integrated anti-tumor effect via immune and inflammatory pathways

The interaction between depression and AE culminates in a combined effect on tumor progression. By ameliorating depressive symptoms, AE indirectly mitigates the chronic inflammatory state and immune dysfunction that fuels tumor growth. Furthermore, AE directly reduces systemic inflammation by modulating immune responses and enhancing anti-tumor activity (100). It has been demonstrated that AE can improve immune function in depressed individuals by lowering pro-inflammatory factors, thereby reducing cancer risk and blocking tumor progression (28, 101). Thus, AE serves as an auxiliary anti-tumor strategy by concurrently tackling depression-driven pathways and directly boosting immune surveillance.

##### 3.3.1.4 Clinical considerations and the imperative for personalization

Despite the overall positive profile, the efficacy of AE is subject to significant individual variation. Factors such as the baseline severity of depression, pre-existing exercise capacity, and the specific type, intensity, and frequency of AE all influence outcomes (32). It is critical to note that extreme AE may induce emotional instability, increase physical burden, and lead to transient immunosuppression, potentially negating its benefits. Therefore, future research must prioritize the development of individualized AE prescriptions that maximize therapeutic benefits for both depression and cancer while avoiding adverse effects.

#### 3.3.2 Dual mechanisms of aerobic exercise in counteracting tumor progression

AE regulates tumor progression through a dual mechanism of action, involving both direct effects on the tumor immune microenvironment and indirect pathways that ameliorate depression-driven chronic inflammation. These interconnected processes operate across molecular, cellular, and physiological levels. **Table 1** outlines the dual regulatory mechanisms of AE in tumor progression.

**Table 1:** Dual regulatory mechanisms of aerobic exercise (AE) in tumor progression: a synthesis at the molecular, cellular, and physiological levels.

Level of action	Specific regulatory mechanisms	Key factors/pathways	Biological effects	References
Molecular Level	1. Regulate neurotransmitter levels to improve neurotransmitter imbalance	5-HT, norepinephrine	Delay chronic inflammatory responses triggered by depression and alleviate depressive symptoms	(102, 103)
	2. Reduce the levels of stress hormones	Cortisol	Inhibit tumor growth and spread	(102, 103)
	3. Activate antioxidant enzymes to reduce oxidative stress	Superoxide dismutase (SOD), catalase (CAT)	Decrease the production of inflammatory factors induced by oxidative stress and regulate inflammatory responses	(30)
	4. Regulate inflammation-related signaling pathways	NF-κB signaling pathway	Inhibit chronic inflammatory responses, optimize the tumor immune microenvironment, and enhance the inhibitory effect of the immune system on tumors	(30, 104)
Cellular Level	1. Enhance immune cell functions	T cells, natural killer cells (NK cells)	Improve T cell activity and NK cell killing function, and strengthen the anti-tumor surveillance role of the immune system	(105)
	2. Regulate macrophage phenotypic transformation	Macrophages (polarization to M1 type)	Enhance the anti-tumor effect of macrophages	(106)
	3. Affect dendritic cell functions (indirectly through antioxidant effects)	Dendritic cells, inflammatory factors	Reduce excessive production of inflammatory factors by dendritic cells under oxidative stress, indirectly inhibiting chronic inflammation	(30)
Physiological Level	1. Enhance blood circulation and increase oxygen supply	-	Delay tumor hypoxia and reduce chronic inflammation triggered by hypoxia	(107)
	2. Improve redox balance	-	Alleviate the negative impact of hypoxia on tumors, optimize the tumor microenvironment, and provide new ideas for tumor treatment	(107)

##### 3.3.2.1 Molecular-level mechanisms: regulating neuro-endocrine-immune crosstalk and oxidative stress

AE orchestrates a multi-system response at the molecular level to counteract the pro-tumorigenic state. Firstly, by modulating neurotransmitter levels (e.g., 5-HT, norepinephrine) and reducing stress hormones (e.g., cortisol), AE alleviates depressive symptoms and subsequently dampens the chronic inflammatory response that promotes tumor growth and spread (102, 103). Secondly, AE activates key antioxidant enzymes, such as superoxide dismutase (SOD) and catalase (CAT), to reduce oxidative stress—a known inducer of inflammatory factor production in immune cells like dendritic cells (30). Thirdly, moderate AE directly inhibits the NF-κB signaling pathway, a master regulator of inflammation, thereby reducing the expression of pro-inflammatory cytokines and creating a less favorable tumor immune microenvironment (30, 104).

##### 3.3.2.2 Cellular-level mechanisms: direct enhancement of anti-tumor immunity

The anti-tumor impact of AE is directly mediated through the functional enhancement of key immune cells. Evidence indicates that AE boosts the activity of cytotoxic T cells and improves the tumor-killing function of natural killer (NK) cells, thereby strengthening the immune system’s surveillance and elimination of malignant cells (105). Furthermore, AE promotes the polarization of tumor-associated macrophages towards the anti-tumor M1 phenotype, enhancing their phagocytic capacity and overall anti-tumor efficacy (106). These cellular changes provide a strong theoretical basis for employing AE as an adjuvant to cancer immunotherapy.

##### 3.3.2.3 Systemic physiological-level mechanisms: improving the tumor microenvironment

Beyond molecular and cellular actions, AE induces beneficial changes in the host’s physiology that indirectly inhibit tumors. By enhancing systemic blood circulation and oxygen delivery, AE alleviates tumor hypoxia—a key driver of malignancy and chronic inflammation within the tumor microenvironment (107). The improvement in oxygen supply and redox balance helps normalize the tumor microenvironment, thereby reducing inflammation and creating conditions less conducive to tumor progression and metastasis (107).

## 4 Conclusion and prospect

### 4.1 Current evidence summary and conversion potential

This narrative review elucidates the mechanism by which depression promotes tumor inflammatory progression through the neuro immune endocrine axis from a molecular and cellular perspective, and demonstrates the enormous potential of aerobic exercise (AE) as a multifunctional non pharmacological intervention in regulating this axis, exerting antidepressant, anti-inflammatory, and anti-tumor effects. Current evidence suggests that integrating AE into cancer treatment, especially for patients with comorbidities of depression, has a solid scientific foundation and promising clinical application prospects. Depression is not just a psychological accompanying state of cancer. It actively shapes a microenvironment conducive to tumor growth, invasion, and immune escape through core biological mechanisms such as neurotransmitter imbalance, HPA axis overactivation, and chronic inflammation. Convergence, AE exhibits unique bidirectional regulatory ability: it can directly act on the tumor immune microenvironment, such as enhancing the cytotoxicity of CD8+T cells and NK cells, promoting macrophage polarization towards M1 phenotype, and reducing key pro-inflammatory factors such as IL-6 and TNF - α; It can also effectively alleviate depressive symptoms by upregulating BDNF, restoring the balance of monoamine neurotransmitters, and reducing cortisol levels. This dual positive regulation of the “body mind axis” has made AE go beyond the traditional scope of supportive therapy and become a core intervention strategy that can simultaneously target the shared pathophysiological mechanisms of cancer and depression. Therefore, systematically integrating it into the psychological oncology and standard cancer care system has urgent clinical needs and enormous translational value.

### 4.2 Integrating AE into clinical routine pathways and challenges

Translating AE from research evidence into clinical practice requires a systematic implementation pathway and overcoming corresponding challenges. Introduce depression and anxiety screening tools (such as PHQ-9, GAD-7) routinely in oncology clinics and simultaneously assess patients’ physical status (such as 6-minute walk test) and comorbidities to identify high-risk populations and establish safe baselines. Adhering to the concept of “exercise is good medicine”, individualized AE prescriptions are issued by trained medical or sports professionals. The prescription should comprehensively consider the type and stage of cancer, the side effects of current anti-tumor treatment plans (such as chemotherapy, radiotherapy, immunotherapy), the patient’s physical fitness level, personal preferences, and the severity of comorbid depression, and dynamically adjust the intensity, frequency, and pattern of exercise (such as considering the combination with resistance training). Build a multidisciplinary team consisting of oncologists, nurses, psychologists/psychiatrists, physical therapists, and certified exercise physiologists. Establish a clear referral pathway (such as from an oncologist to a sports rehabilitation center) to ensure that patients receive coherent and professional guidance. Develop tumor rehabilitation centers within hospitals, projects in collaboration with community fitness institutions, and home-based wearable device remote monitoring solutions to meet the accessibility and needs of different patients The main challenges and countermeasures include the lack of a unified medical insurance reimbursement policy, insufficient knowledge of medical staff’s time and exercise prescription, compliance difficulties caused by treatment side effects (such as severe fatigue), and concerns about exercise safety. We need to promote the inclusion of tumor sports rehabilitation in medical insurance at the policy level; Strengthen the continuing education of oncology medical staff; Develop safe exercise guidelines and standard operating procedures for different types of cancer and treatment stages; Utilize behavior change techniques (such as motivational interviews) and digital health tools to enhance long-term patient compliance.

### 4.3 Future research directions and key knowledge gaps

In order to fully leverage the potential of AE in tumor treatment, future research should focus on filling the following key knowledge gaps: in the future, it is necessary to use preclinical models and human biological samples collected from interventional clinical trials (such as blood and tumor tissue) to explore in depth the specific mechanisms by which AE regulates neural plasticity, immune cell metabolism (such as T cell mitochondrial function), and epigenetic remodeling in depression cancer comorbidity, in order to discover new biomarkers and therapeutic targets. More head-to-head randomized controlled trials are urgently needed to determine the optimal AE parameters (pattern, intensity, dose, and timing) for specific cancer types (such as immune “cold” and “hot” tumors), specific treatment methods (such as immune checkpoint inhibitor therapy), and specific patient subgroups (such as elderly, advanced, and severe cachexia). Currently, most research focuses on alternative endpoints such as symptoms and quality of life. In the future, large-scale, long-term follow-up studies must be designed and implemented to ultimately confirm the substantial impact of regular adverse events on cancer recurrence rates, progression free survival, and overall survival. The combination of AE and emerging anti-cancer therapies such as immunotherapy and targeted therapy is a highly promising direction. Research should focus on how AE can reverse the immunosuppressive microenvironment by improving tumor angiogenesis, alleviating hypoxia, enhancing immune cell infiltration, and other functional changes, thereby increasing sensitivity and overcoming treatment resistance. By utilizing wearable devices, mobile medical apps, and artificial intelligence algorithms, objective and continuous monitoring of patients’ daily activity levels, exercise compliance, and physiological parameters can be achieved. This not only promotes the dynamic adjustment of personalized exercise prescriptions but also provides an unprecedented opportunity to study the “real-world” exercise effects in a large sample population.
